# Report of a Case of Idiosyncrasy in Regard to the Effects of Quinine

**Published:** 1877-04

**Authors:** E. M. Dening


Report of a Case of Idiosyncrasy in Regard to the Effects of Quinine.—*By E. M. Dening, M.D.*—(*Read before the Pathological Society of Columbus, Ohio.*)—In the summer of 1862 was called to see a boy about seven years of age. From his symptoms I supposed he was suffering from a slight attack of intermittent fever, and prescribed quinine. On seeing him again, in the evening of the same day, I was somewhat surprised to find his whole body covered with an apparently scarlatinous eruption, accompanied with intense itching, some fever and general restlessness. Supposing now that I had a case of scarlet fever to deal with instead of intermittent, the quinine was suspended, and the other treatment resorted to. The next day the rash disappeard and the boy recovered without much medication.

Two years subsequently he had a similar attack, which in my mind indicated the use of quinine. and without suspecting its agency in producing the symptoms referred to, we again administered it. The effects which followed were precisely similar to the former occasion, only somewhat intensified.

In twenty minutes after taking the quinine he became red from head to foot, with intense itching over the whole surface; his agitation was great, face swolen, eyes red and suffused, breathing difficult, presenting, altogether the appearance of extreme suffering.

I now became satisfied he had some idiosyncrasy or peculiarity which induced these attacks of urticaria when quinine was administered, and so stated, warning his parents against giving it or allowing any other person to do so, under any circumstances.

In 1864, being absent from the city, this lad was again taken very sick.

The gentleman in whose care I had left my business, called to see him.

Tho mother of the boy, fearing the doctor might possibly prescribe quinine, explained to him its peculiar effects, and stated that I had cautioned them against its use.

The incredulous smile of the doctor led her to assure him that she had no foolish prejudices against the use of the medicine in question; that they always purchased it by the ounce and were accustomed to use it freely, with and without medical advice.

He made no reply, but wrote his prescription. In a few hours he was again summoned to the house and found the boy in a most alarming condition, all the symptoms we have enumerated being well pronounced. The doctor, on seeing the state of things, was seriously alarmed for the boy.

He was assured, however, by the parents, that the worst was over. On my return home he gave me an account of the case, stating how much he had been frightened, adding, that in all his long experience he had never witnessed similar symptoms, nor apparently greater suffering, finishing by requesting me to report the case.

A few years later this now young man, after having visited Rome, was attacked, on reaching Paris, with what is called Roman Fever. The medical gentleman to whom he applied again prescribed quinine, which he took without suspecting its presence. He was not long, however, in realizing, from the effects produced, that the prescription contained his now too well recognized foe.

Some six or eight years again elapsed, when he had an attack of fever of well marked malarial type, and of more than usual severity.

Knowing but too well his inability to take the appropriate remedy, I determined on trying salicin; but owing to unaccountable misunderstanding he again took five grains of quinine. This was taken during the period of intermission, when he was feeling quite comfortable.

In ten minutes after swallowing it he felt the effects in every fibre of his body; his face became red, his lips and eyelids swollen, and whole features bloated. The effect upon the respiratory passages which soon followed, resembled a violent attack of asthma or hay fever; the posterior nares and trachea seemed swollen to an extent as almost to preclude the access of air, the threatened asphyxia compelling him to breath with his mouth wide open and *alae nasi* unduly distended.

No language could describe the apparent anguish and distress present; every muscle which could exert influence on the respiratory function was brought into violent requisition; his agitation was extreme, compelling him to keep in motion; his bloodshot, eyes and swollen features giving him the most frightful appearance.

After some hours of suffering such as we have endeavored to describe, the secretion of tears became abundant, a profuse discharge from the nostrils occurred, and the symptoms began to subside.

He did not, however, recover his wonted appearance for some days, the itching still persisting to a great extent, accompanied by a weary and exhausted feeling.

The singularity of the case made a strong impression on my mind, as it seems to have done on that of my confrere. I had never witnessed before like results following the administration of quinine, nor seen any case of the kind reported.

No allusion is made in the works on Materia Medica which I have examined to these peculiar effects.

In the October number, however, of the *Annals of Hygiene*, published in Paris, I do find a case reported which is, in every respect, the exact fac simile of mine.

It was meeting with this reported case—the exact resemblance of the two cases—which recalled my attention to the matter, and has induced me to give both the history of my own case and a translation of the case referred to, thinking the matter may not be devoid of interest to at least some member of this Society.

Dr. Adolph Dumas, Adjunct Surgeon of the Hospital de Cetti, says: A fémale, a distant relation of mine, has for some time presented a singular special sensibility to the action of quinine. Five times in a year, at intervals of several months, she has experienced these symptoms which, on the last occasion, have assumed alarming proportions. The following details of the case I have collected from observation made at the bedside of the patient.

In consequence of a facial neuralgia to which this woman is subject, I ordered her, on the 20th of July, seven P.M., to take two pills containing five grains of quinine.

Owing to an impression which she had received from former experience, that the effects were less unpleasant when not taken on an empty stomach, she was directed to take a slight repast before taking the quinine.

Ten minutes had scarcely elapsed after its ingestion, when she was seized with extreme restlessness and agitation, flushing of the face, tension of the eyelids, and a prickling sensation around the mouth. In ten minutes there was vomiting of part of the porridge she had taken, and judging from its bitterness a portion of the quinine, accompanied with slight oppression of breathing.

By degrees all these symptoms were aggravated: oppression quite strong; respiration hurried; vivid injection of the face and eyelids, which became distended and half closed; eyes red and watery; intense itching of the whole body, especially where there was any constriction by the clothing, which compelled her to undress and go to bed.

I can distinguish, in some places, the elevated urticarious eruption; but generally there is redness without elevation, resembling the scarlatinous eruption; in some places, however, there are real papules which render the part rough to the touch. These latter progressively augmented. The patient scratches herself furiously, excoriating the body in several places; the traces of the nails being at first white, but soon becoming red and remaining so; the agitation is extreme, not a part of her person being free from pungent itching. She suffers much in the palms of the hand and between the fingers and toes.

The itching is much increased by the least change of posture.

The oppression becomes greater and greater; respiration panting and sibilant, which can be heard at a distance, and is seventy-two per minute; pulse 104; temperature, 101.8; has a little dry cough. She expresses, at times, a feeling of impending suffocation, rejecting all covering, although she is not hot. There are moments when her agony is almost unendurable, and at no time does it entirely relent.

A little later the secretion of tears is augmented, and abundant discharge flows from the nostrils, with freqent paroxysms of sneezing.

Eleven o'clock.—Seven hours after taking the quinine the acute suffering commences to diminish, the itching still remaining. I should add that as soon as the symptoms here described were fully developed, the neuralgic paines ceased to a great extent until eleven o'clock, the time at which the habitual exascerbation occurred.

Twelve o'clock.—The coryza, itching, and oppression diminishing in intensity, and the patient commenced to have some moments of repose, interrupted by an occasional exascerbation, especially of the oppressive breathing.

The patient seemed much exhausted and kept her bed for several days, during which her skin, usually soft and delicate, remained rough and furfuraceous.

This was the fourth time, as I observed in the commencement, that I had witnessed a similar effect produced on this young woman from the administration of quinine.

In the first instance the dose being very small (about two grains,) the symptoms, although of the same character, were not so violent, and lasted only about an hour. Being quite unprepared for such a result, I asked myself whether the quinine could be the cause, and as she was quite well the next day, another pill containing the same amount was given, with precisely the same result.

As the patient charged her symptoms to the drug administered, she was unwilling to again resort to its use; to test how much might be due to imagination, I had it administered without her knowledge; the effects were soon over. Three times, the amount taken was only two grains, and on the fourth and last occasion only five grains. It is obvious that the effects produced by the quinine were in proportion to the amount prescribed, as in the last instance they were truly alarming and distressing, although, in this case, a portion of the medicine had been ejected by vomiting.

What would the result have been had a much larger dose been administered? It is probable, however, that the dose here is only of secondary importance, and that the effects are due to the peculiar idiosyncrasy of the individual almost exclusively. In a severe attack of fever which she experienced at that period, eight grammes of the sulphate of quinine were given in divided doses without producing anything but the ordinary effects of the drug.

The accession of the phenomena above described has always been the same, appearing first on the face, lips and eyes; countenance bloated, eyelids swollen, and eyes suffused; following in rapid succession is the intolerable itching of the surface, eruption, and general feeling of tension; the mucuous membrane soon participating in the disturbance, producing rapid and oppressed breathing—a real attack of asthma. A little later a discharge takes place from the nostrils, abundant tears flow from the eyes, the breathing improves, the nervous agitation is less violent, and the patient, exhausted and weak, gradually regains her usual health.—*Ohio Med. Recorder.*

